# Supplementary material for: The causal relationship between the human gut microbiota and pyogenic arthritis: a Mendelian randomization study
Source: Front Cell Infect Microbiol. 2024 Nov 26;14:1452480. doi: 10.3389/fcimb.2024.1452480 (PMC11629706; doi:10.3389/fcimb.2024.1452480)
Supplement: Supplementary file 1 [file DataSheet1.zip › mendelian test/ebi-a-GCST90027752.csv_forest.pdf]

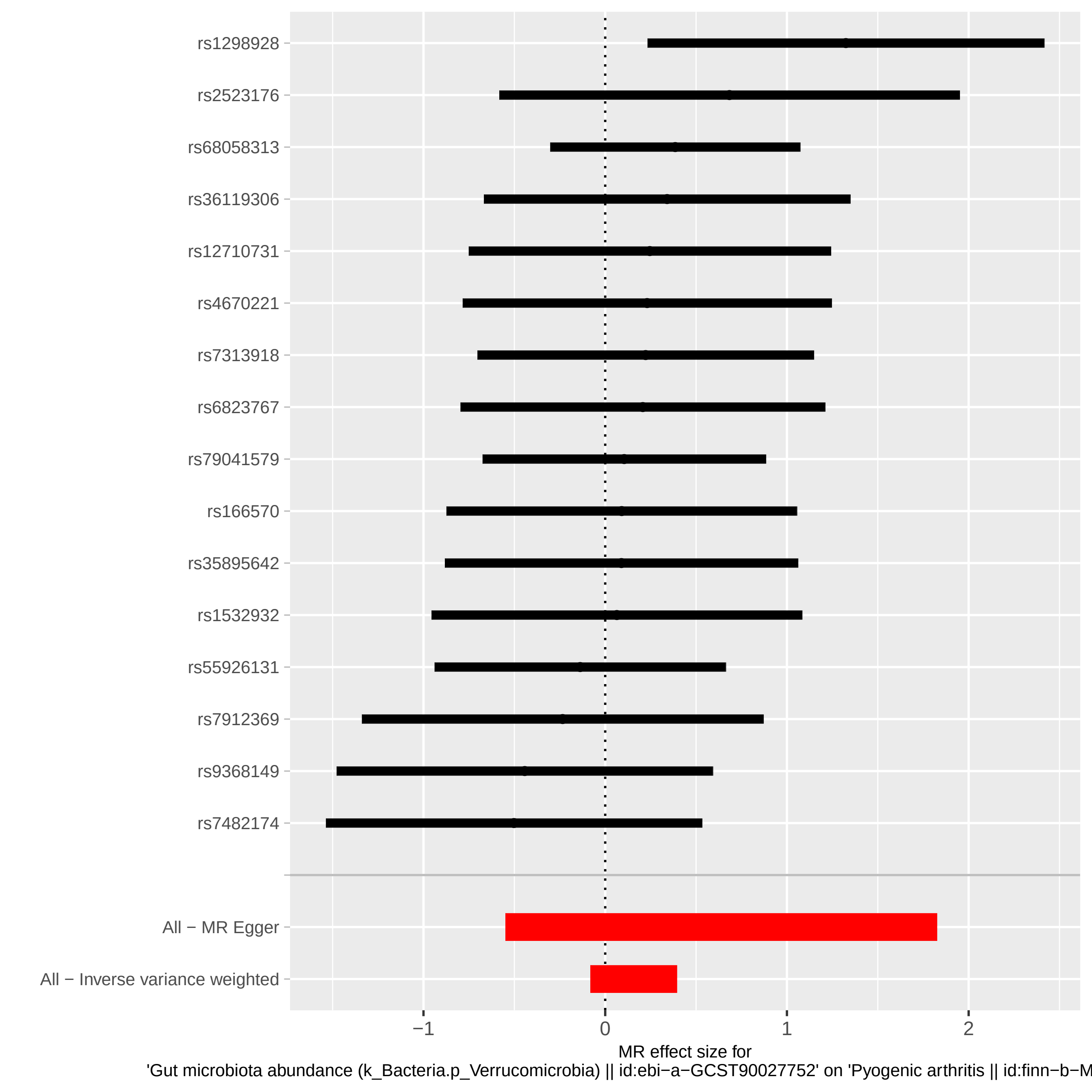
rs1298928
rs2523176
rs68058313
rs36119306
rs12710731
rs4670221
rs7313918
rs6823767
rs79041579
rs166570
rs35895642
rs1532932
rs55926131
rs7912369
rs9368149
rs7482174
All − MR Egger
All − Inverse variance weighted
−1
0
1
2
MR effect size for
'Gut microbiota abundance (k_Bacteria.p_Verrucomicrobia) || id:ebi−a−GCST90027752' on 'Pyogenic arthritis || id:finn−b−M
